# Visual information density in AR commerce: cognitive responses to professional sport jerseys

**DOI:** 10.3389/fpsyg.2026.1882598

**Published:** 2026-06-26

**Authors:** Jun-Phil Uhm, Seungmin Lee

**Affiliations:** 1 Department of Kinesiology, Inha University, Incheon, Republic of Korea; 2 Institute of Coaching, Waseda University, Tokyo, Japan

**Keywords:** AR commerce, arousal, attention, augmented reality, cognitive processing, perceptual engagement, visual clutter, visual information density

## Abstract

**Introduction:**

Augmented reality (AR)–based commerce environments increasingly encourage users to actively inspect visually complex products rather than passively view them. In sport merchandize contexts, professional team jerseys containing multiple sponsor logos represent visually dense products that may shape perceptual and cognitive responses during immersive product evaluation. This study examines how variations in visual sponsor logo density on professional team jerseys are associated with perceived visual clutter, attention, arousal, and attitudes toward the jersey in an AR-based shopping environment.

**Methods:**

A between-subject experimental design was employed in which 78 participants viewed professional baseball jerseys containing either low or high levels of sponsor logo density within an AR shopping environment. The data were analyzed using analysis of variance.

**Results:**

Jerseys containing higher sponsor logo density generated significantly greater perceptions of visual clutter and produced significant differences in attention and arousal. No statistically significant differences emerged for attitudes toward the jersey.

**Discussion:**

The findings suggest that increased visual logo density primarily influences perceptual and cognitive processing during immersive AR-based product interaction, whereas overall evaluative impressions toward the product remain comparatively stable. The results further indicate that visually saturated product designs may shape experiential engagement more readily than broader attitudinal responses in immersive digital commerce contexts.

## 1 Introduction

Immersive commerce technologies are increasingly transforming how consumers interact with products in digital retail environments. In particular, augmented reality (AR)–based shopping systems allow users to actively manipulate, inspect, and evaluate products through real-time immersive interaction rather than through passive exposure to static displays (Javornik, 2016; Yim et al., 2017). Compared with conventional online retail formats, AR environments encourage more sustained and goal-directed product exploration, thereby increasing the relevance of perceptual and cognitive processing during product evaluation (Uhm et al., 2022). As immersive retail technologies continue to expand across commercial sectors, understanding how visually complex product designs shape users’ perceptual and evaluative experiences has become an increasingly important issue within AR-mediated commerce environments (Sun et al., 2025). Recent industry developments additionally suggest growing implementation of AR-based commerce systems and interactive product visualization technologies across digital retail sectors (Sandhiya, 2025; Varsha, 2025).

Research concerning visual complexity and attentional processing suggests that visually dense displays may influence how individuals allocate limited cognitive resources when interacting with products containing multiple competing visual elements (Ha and McCann, 2008; Im et al., 2021; Lang, 2000; Pieters et al., 2002). Prior research on visual clutter further indicates that crowded visual environments can increase perceptual complexity and interfere with efficient information processing when numerous visual cues compete simultaneously within limited visual space (Guo and Chen, 2023; Rosenholtz et al., 2007). Within immersive AR environments that encourage active product manipulation and sustained inspection, visually information-dense products may therefore impose greater cognitive processing demands and alter users’ attentional and affective responses during evaluation (Buchner et al., 2022).

Professional sport jerseys provide a commercially relevant context for examining these perceptual dynamics because sponsor logos are increasingly integrated into the visual structure of team apparel (Son and Williams, 2023). Across professional sport leagues such as Major League Soccer (MLS) and Major League Baseball (MLB), expanded sponsorship placements have increased the amount of commercial information embedded within jersey designs. Industry reports further indicate that sponsorship integration in professional baseball has expanded substantially in recent years following policy changes permitting additional sponsor placements on uniforms (Brown, 2025; SponsorUnited, n.d.). As sponsor cues accumulate across different regions of the garment, jerseys increasingly function as visually information-dense products containing multiple co-occurring visual stimuli. Although prior sponsorship and advertising literature has suggested that such environments may intensify message competition and perceptions of clutter (Ha and McCann, 2008), comparatively limited research has examined how visually dense sponsor configurations are processed when products are actively inspected within immersive AR retail environments.

The growing integration of AR technologies into sport merchandize retail further heightens the relevance of these issues. Sport organizations and apparel brands have increasingly implemented AR-based product visualization systems and virtual try-on technologies that allow consumers to inspect jerseys through immersive interaction prior to purchase decisions (Bagley, 2024; Goebert, 2020; Snap Inc., 2025). Research in retail and marketing contexts conceptualizes AR as an interface that integrates digital information with users’ physical surroundings, thereby increasing interactivity and personal relevance during product evaluation (Javornik, 2016). Existing evidence additionally suggests that AR-based shopping experiences encourage more deliberate exploration of product attributes and may shape experiential and evaluative responses during digital retail interaction (Uhm et al., 2022). Within these immersive environments, consumers are able to rotate, enlarge, and closely inspect visual product features over extended periods of time, potentially increasing sensitivity toward structural design characteristics such as sponsor logo density.

Recent AR retail research further suggests that emotional responses generated during immersive shopping experiences may play an important role in shaping consumer outcomes. For example, Gary et al. (2025) found that emotional responses elicited during AR experiences contribute to e-commerce consumption decisions, highlighting the potential importance of affective processes during product evaluation. Similarly, Soon et al. (2023) reported that AR-induced emotions can significantly influence consumers’ willingness to engage with AR technologies and shape subsequent behavioral responses within digital retail environments. Consequently, examining emotional responses such as arousal may provide additional insight into how consumers react to product characteristics within immersive retail environments.

Research on attentional capacity and cognitive processing provides additional theoretical support for understanding these perceptual mechanisms. Cognitive Load Theory (CLT) proposes that individuals possess limited cognitive processing capacity and may experience increased processing demands when confronted with visually complex or information-rich stimuli (Sweller, 1988). Similarly, the Stimulus–Organism–Response (S–O–R) framework suggests that environmental stimuli can evoke internal perceptual and affective states that subsequently shape evaluative responses (Eroglu et al., 2003; Mehrabian and Russell, 1974). Applied to immersive AR commerce environments, these perspectives suggest that visually dense sponsor configurations may influence how consumers cognitively and perceptually process products during interactive evaluation (Chiang et al., 2022; Voicu et al., 2023). Rather than advancing directional predictions, the present study utilizes these frameworks to interpret variations in perceptual and evaluative responses observed during immersive AR-based jersey inspection.

Despite the increasing adoption of immersive retail technologies, limited empirical research has examined how visually dense sponsor configurations influence users’ perceptual and cognitive responses during AR-mediated product evaluation. Existing sponsorship research has primarily focused on passive exposure contexts such as live broadcasts and event viewing environments, emphasizing sponsor recall and attitude formation (Herold and Breuer, 2023; Schönberner and Woratschek, 2022). Comparatively less attention has been devoted to understanding how consumers cognitively engage with sponsor-rich product designs when merchandize is actively explored through immersive AR interaction. Consequently, limited evidence exists regarding whether increased sponsor logo density enhances or disrupts perceptual and evaluative experiences during immersive product inspection. Addressing this issue is increasingly important as sport organizations continue integrating AR technologies into digital merchandize retail systems.

Accordingly, the current study examines how sponsor logo density on professional team jerseys is associated with consumers’ perceptual and evaluative responses during immersive AR-based product inspection. Although AR provides the technological context for product evaluation, the primary focus of the present study is on how variations in visual information density are perceived and processed by consumers. More broadly, the study seeks to contribute to user-centered design considerations in immersive commerce environments by examining how visual information density shapes perceptual engagement during interactive product evaluation. Specifically, the study investigates whether variations in sponsor logo density influence perceived visual clutter, attention, arousal, and attitudes toward jerseys within an AR shopping environment. The study is guided by the following research questions:

RQ1: How does sponsor logo density influence consumers’ evaluative processes when professional team jerseys are inspected in an AR–based shopping environment?

RQ2: How does sponsor logo density influence consumers’ arousal and attention during AR-based jersey inspection?

RQ3: How does sponsor logo density influence consumers’ attitudes toward the jersey following AR-based inspection?

## 2 Materials and methods

### 2.1 Research design

The current study employed a between-subjects experimental design to examine how variations in visual sponsor logo density on professional sport jerseys influence perceptual and evaluative responses within an immersive AR-based shopping environment. Participants were randomly assigned to inspect one of two jersey conditions that differed in sponsor logo density: a low-density condition or a high-density condition.

The two jersey stimuli represented different levels of visual information density while maintaining consistency across all other visual characteristics, including team identity, color scheme, jersey structure, and AR presentation interface. This design enabled systematic examination of whether differences in visual sponsor density were associated with distinct perceptual and cognitive responses during immersive AR-mediated product evaluation.

The primary response dimensions examined in the study included perceived visual clutter, arousal, attention, and attitude toward the jersey. Perceived visual clutter was included to verify whether variations in sponsor logo density were perceptually salient during immersive product inspection without presupposing the direction of subsequent evaluative responses.

### 2.2 Participants

The study protocol was approved by the Institutional Review Board (IRB) of the authors’ affiliated institution, and all participants provided informed consent prior to participation. Recruitment targeted adults residing in an East Asian country who were at least 18 years of age. To ensure appropriate participation within the immersive AR environment, eligibility criteria required participants to report corrected-to-normal vision, no diagnosed neurological or cognitive impairments, no sensitivity to digital motion sickness, and no prior experience with AR-based e-commerce systems. Individuals who reported exclusion-related conditions during screening were removed prior to participation.

A total of 81 individuals were initially recruited for the study. Three participants were excluded due to incomplete survey responses or visual discomfort experienced during the AR task, resulting in a final sample of 78 participants. Participants were randomly assigned to either the low-density or high-density sponsor logo condition, with 39 individuals allocated to each group. An *a priori* power analysis conducted using G*Power indicated that a minimum sample size of 48 participants was required to detect a medium effect size (*f*^2^ = 0.25) with statistical power of 0.80 at *α* = 0.05, suggesting that the final sample size was sufficient for the planned analyses. The mean age of participants was 25.32 years, including 46 males and 32 females. Participants reported engaging in general online shopping activities approximately three to four times per month on average, although none had prior experience using immersive AR shopping applications.

### 2.3 Measurement

All study constructs were measured using previously validated scales that demonstrated acceptable reliability and validity in prior research. Unless otherwise specified, all items were assessed using seven-point response scales. To ensure cross-cultural equivalence, the survey instrument was translated using a back-translation procedure (Brislin, 1970).

Perceived visual clutter was assessed using six items adapted from Lewis and Sauro (2024) and related visual clutter research. The scale measured the extent to which the jersey design appeared visually crowded, overloaded, or difficult to process because of the concentration of visual elements. Consistent with prior visual clutter research, the measure was intended to capture participants’ subjective perceptions of visual excessiveness and disruption rather than an objective count of visual elements. Arousal was measured using five items adapted from the arousal dimension of the Pleasure–Arousal–Dominance (PAD) framework (Mehrabian and Russell, 1974), capturing participants’ perceived levels of stimulation and activation while interacting with the jersey within the immersive AR environment.

Attention during immersive jersey inspection was measured using three items adapted from Thorson et al. (1992) and Uhm et al. (2021). These items assessed participants’ perceived attentional focus toward the jersey and its visual characteristics throughout the AR interaction experience. Attitude toward the jersey was measured using a three-item semantic differential scale adapted from MacKenzie and Lutz (1989), capturing participants’ overall evaluative impressions of the product following immersive inspection. The complete wording of all measurement items is presented in the Appendix.

### 2.4 Experimental stimuli

An immersive AR-based product evaluation environment was developed specifically for the present study to simulate an interactive digital shopping context. The AR content was presented using Apple Vision Pro, allowing participants to inspect a three-dimensional representation of a professional baseball jersey within an immersive visual environment. Consistent with contemporary AR commerce interfaces, participants were able to naturally manipulate the product during inspection through behaviors such as rotating, enlarging, and repositioning the jersey within the AR space.

Two jersey versions were created to operationalize differences in visual sponsor logo density. In the low-density condition, the jersey displayed only the team logo and manufacturer logo, resulting in a relatively simple and visually restrained appearance. In contrast, the high-density condition included five sponsor logos distributed across the torso and sleeve regions of the jersey, creating a visually information-dense presentation characteristic of sponsor-rich apparel designs. All remaining visual characteristics, including team identity, color scheme, and jersey structure, were held constant across conditions to isolate the effects of sponsor logo density during immersive product inspection.

**Figure 1:**
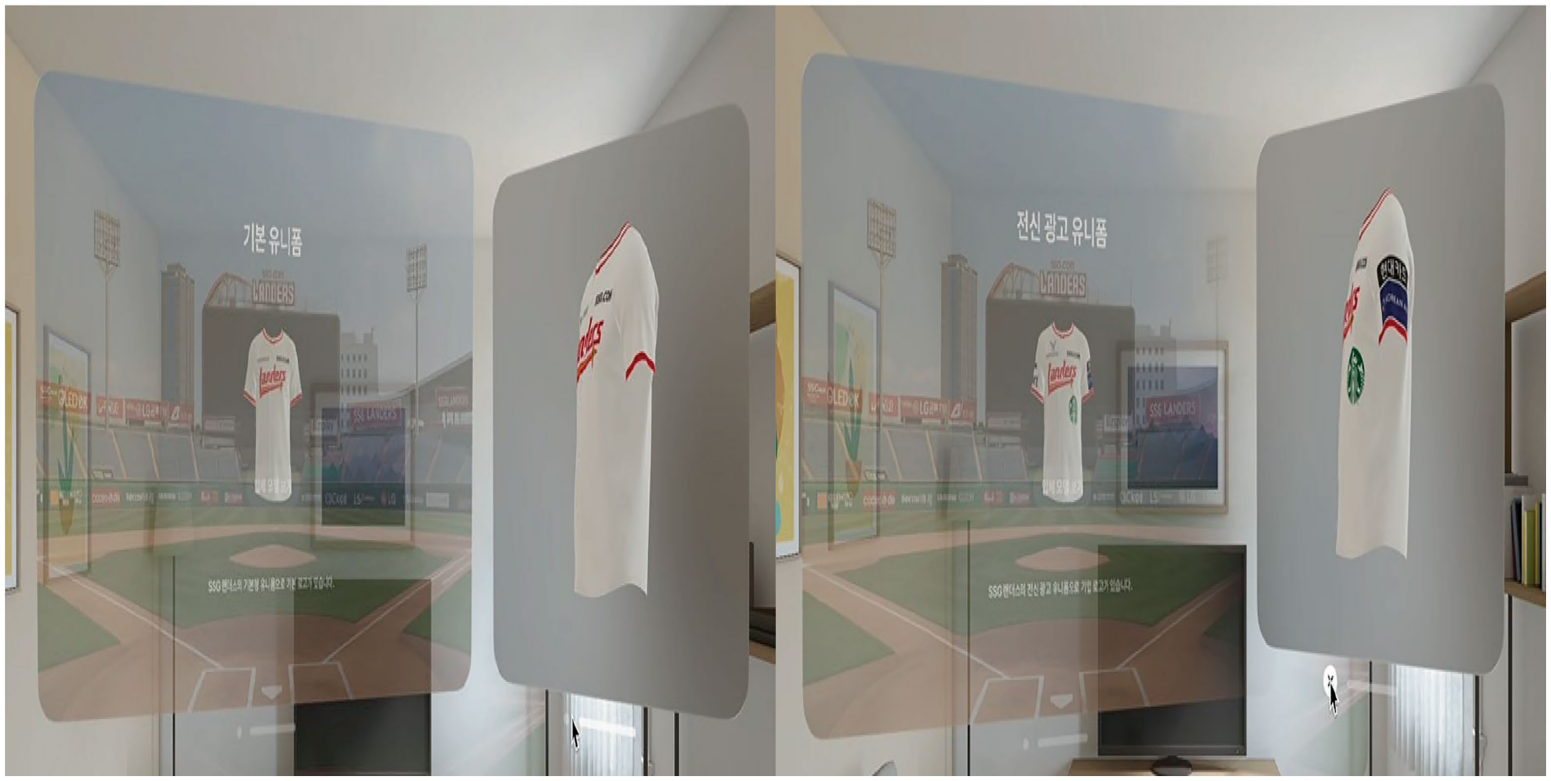
Jersey stimulus displaying sponsor logo density.

To enhance ecological validity, participants viewed jerseys associated with professional baseball teams they identified as familiar prior to the experiment. Official team visual identities were consistently applied across both density conditions to support realistic product evaluation and minimize novelty effects associated with unfamiliar designs. Figure 1 presents the jersey stimuli used in the study.

### 2.5 Procedure

Upon arrival at the experimental site, participants received an explanation of the study procedures and provided written informed consent. Participants then completed a brief pre-experimental questionnaire that collected demographic information and identified the professional baseball team with which they were most familiar. This information was subsequently used to personalize the jersey stimulus presented during the immersive AR experience.

Participants were then fitted with an AR head-mounted display that was individually calibrated to ensure visual clarity and proper fit. Prior to the immersive task, a brief screening procedure was conducted to identify any potential discomfort associated with AR exposure. Participants were randomly assigned to one of the experimental conditions and instructed to inspect the jersey as though they were evaluating it for potential purchase. During the immersive AR interaction, participants were free to rotate, enlarge, and reposition the jersey model according to their preferred inspection behaviors.

Following completion of the immersive inspection task, participants completed a post-experience questionnaire assessing perceived visual clutter, attention, arousal, and attitude toward the jersey. After the experiment concluded, participants were debriefed and compensated with a small coffee voucher equivalent to approximately USD $7.

### 2.6 Data analysis

All statistical analyses were conducted using IBM SPSS Statistics 31.0. Preliminary analyses included descriptive statistics, reliability assessment, correlation analysis, and multicollinearity diagnostics, with the results summarized in Table 1. Descriptive statistics were first examined to assess data quality and distributional assumptions. Skewness and kurtosis values for all variables remained within recommended thresholds (±2 for skewness; ±7 for kurtosis), indicating no substantial violations of normality assumptions (West et al., 1995).

**Table 1:** Descriptive and correlational statistics of research variables.

Variable	1	2	3	4
1. Perceived clutter	–			
2. Arousal	0.241*	–		
3. Attention	0.112	0.206	–	
4. Attitude	−0.104	−0.316**	−0.082	–
*M*	4.252	4.049	3.692	3.410
*SD*	0.675	1.214	1.276	0.793
Skewness	0.778	−0.223	−0.099	−0.692
Kurtosis	2.787	0.993	0.290	−0.537
Cronbach’s *α*	0.768	0.824	0.867	1.043

*p < 0.05, **p < 0.01.

Internal consistency reliability was evaluated using Cronbach’s *α* coefficients, and all constructs exceeded the recommended criterion of 0.70 (Nunnally and Bernstein, 1994). Pearson correlation coefficients were additionally examined to assess associations among variables and identify potential multicollinearity concerns, with all correlations remaining below 0.85 (Kline, 2016).

To evaluate the effectiveness of the sponsor logo density manipulation, a one-way analysis of variance (ANOVA) was conducted using perceived visual clutter as the dependent variable. To address the study’s research questions concerning perceptual and evaluative responses during immersive AR-based product inspection, separate ANOVAs were conducted with sponsor logo density (low versus high) as the independent variable. Attention, arousal, and attitude toward the jersey were examined as dependent variables in separate models. This analytical approach enabled assessment of whether differences in visual sponsor density were associated with distinct perceptual and evaluative responses during immersive AR interaction.

## 3 Results

### 3.1 Manipulation test

A one-way analysis of variance (ANOVA) was conducted to determine whether the two experimental conditions differed in perceived visual clutter during immersive AR-based jersey inspection. The analysis revealed a significant difference in perceived visual clutter between the low-density and high-density sponsor logo conditions (*F*(1, 76) = 18.204, *p* < 0.001, *η*^2^ = 0.193). Participants exposed to the high-density jersey condition reported significantly greater levels of perceived visual clutter (*M* = 4.547, *SD* = 0.671) than those assigned to the low-density condition (*M* = 3.957, *SD* = 0.543). These findings indicate that variations in visual sponsor logo density were perceptually salient within the immersive AR evaluation environment, supporting the effectiveness of the experimental manipulation.

### 3.2 Main analyses

To examine whether variations in sponsor logo density were associated with differences in perceptual and evaluative responses during immersive AR-based product inspection, separate ANOVAs were conducted for attention, arousal, and attitude toward the jersey across the two experimental conditions. Prior to analysis, the assumption of homogeneity of variance was assessed, and no significant violations were identified. Collectively, these analyses were conducted to evaluate how differences in visual sponsor density were associated with distinct cognitive, perceptual, and evaluative response dimensions during immersive product interaction.

As presented in Table 2, the results indicated that sponsor logo density was associated with differential response patterns across the examined outcome variables. Specifically, participants in the high-density condition reported significantly higher levels of arousal during immersive jersey evaluation, whereas participants in the low-density condition demonstrated significantly higher levels of attention. By contrast, no statistically significant differences emerged between conditions with respect to overall attitudes toward the jersey following the AR-based inspection experience.

**Table 2:** Summary of ANOVA results.

Dependent variable	Low density *M* (*SD*)	High density *M* (*SD*)	*F*(1, 76)	*p*	*η* ^2^
Arousal	3.713 (1.286)	4.385 (1.049)	6.391	0.014	0.078
Attention	4.128 (1.215)	3.256 (1.191)	10.238	0.002	0.119
Attitude	3.418 (0.748)	3.402 (0.845)	0.009	0.925	<0.001

Homogeneity of variance was satisfied for all dependent variables (Levene’s test, *p*s > 0.05).

## 4 Discussion

### 4.1 General discussion

The findings of the present study suggest that variations in sponsor logo density, as a form of visual information density, systematically influence perceptual and cognitive responses during immersive AR-based jersey evaluation. As a visually embedded product characteristic, sponsor logo density altered the overall visual complexity of the jersey by increasing the concentration of simultaneously presented commercial cues. The manipulation test confirmed that participants consistently perceived higher sponsor logo density as greater visual clutter, indicating that the intended differences in visual information density were successfully recognized during immersive inspection. Building upon this perceptual distinction, the observed attention effects appear to reflect consumers’ cognitive responses to visually dense product configurations rather than broader evaluative judgments toward the jersey itself (Paas and van Merriënboer, 1994; Pieters and Wedel, 2004).

From a theoretical perspective, Cognitive Load Theory (CLT) provides a useful framework for interpreting these attentional patterns. CLT proposes that individuals possess limited cognitive processing capacity and may experience increased processing demands when multiple information elements must be processed concurrently (Sweller, 1988). Within the present immersive AR environment, higher sponsor logo density increased the perceptual complexity of the jersey and heightened perceptions of visual clutter during interactive inspection. This increase in perceived complexity likely elevated cognitive processing demands, thereby influencing participants’ attentional states throughout the evaluation process (Paas and van Merriënboer, 1994). Given the operationalization of the construct, attention in the present study reflects participants’ subjective perception of attentiveness during jersey inspection rather than an objective measure of visual attention or attentional allocation (Lang, 2000; Lavie, 2005). Although the immersive AR environment facilitated detailed product inspection, the attentional differences observed across conditions appear to have been primarily driven by variations in the visual structure of the jersey design itself. Importantly, the present findings should be interpreted within the context of AR-based product evaluation rather than as evidence of effects unique to AR technology itself.

Taken together, the observed pattern of lower attention and higher arousal suggests that sponsor-rich jerseys may have created a more stimulating yet cognitively demanding inspection experience, thereby helping explain the divergent responses observed across the two constructs. Rather than representing contradictory responses, these findings may reflect a state of perceptual overstimulation in which the accumulation of multiple visual elements increased the overall sensory intensity of the AR experience while simultaneously making it more difficult for participants to maintain focused attentiveness toward the jersey (Berlyne, 1960; Eroglu et al., 2003). In addition, if participants experienced these visually dense displays as overwhelming, the resulting activation may have functioned as a distractor, thereby reducing their ability to maintain focused attentiveness toward the jersey. Although the present study did not directly assess frustration, stress, or other negative affective states, the findings may nevertheless be consistent with the possibility of visual overload resulting from visually dense sponsor configurations.

The findings additionally demonstrated that sponsor logo density was associated with significant differences in arousal during immersive AR-based product interaction. Jerseys containing higher levels of sponsor logo density produced greater arousal responses, suggesting that visually information-dense product designs increased the experiential intensity of the inspection experience. Even though participants were not required to make explicit purchase decisions or comparative evaluations, increased visual complexity within the immersive AR environment appeared to heighten perceptual activation during product exploration.

These arousal differences may be explained by the influence of visually dense environments on cognitive activation during immersive interaction. Prior research in environmental psychology and advertising has suggested that increased concentrations of visual stimuli elevate perceptual load and sensory stimulation, which may subsequently heighten arousal responses (Eroglu et al., 2003; Li et al., 2002; Mehrabian and Russell, 1974). Within the present AR-based shopping context, sponsor-rich jersey designs increased the overall visual intensity of the immersive product display and were perceptually experienced as more cluttered environments. This heightened visual intensity may have amplified cognitive activation as participants actively manipulated and explored the jerseys through immersive AR interaction. Importantly, arousal in this context reflects the magnitude of cognitive and perceptual activation rather than the positive or negative direction of evaluative judgment (Berlyne, 1960; Mehrabian and Russell, 1974).

By contrast, no significant differences emerged in overall attitudes toward the jersey across sponsor logo density conditions. One possible explanation relates to the nature of the immersive evaluation context employed in the present study. Participants engaged in relatively brief exploratory interaction with a single product and were not required to make explicit comparative judgments or purchase-related decisions during the AR experience. Prior literature suggests that broader product attitudes may remain relatively stable following isolated exposure episodes, particularly when individuals are not prompted to engage in deliberate evaluative decision-making processes (Petty and Cacioppo, 1986; Zajonc, 1968). Under these conditions, temporary variations in perceptual processing, attention, or arousal may remain confined to the experiential level rather than extending into broader attitudinal evaluations (Lang, 2000; MacKenzie et al., 1986).

Recent AR retail research suggests that emotional responses generated during immersive experiences can contribute to downstream consumer outcomes (e.g., Gary et al., 2025; Soon et al., 2023). However, the present study found no significant differences in attitudes toward the jersey. This finding suggests that heightened arousal alone may not be sufficient to influence broader product evaluations, particularly when consumers engage in a brief exploratory task, exhibit relatively low involvement with the evaluated product, and are not required to make explicit purchase-related decisions. It is also worth noting that mean attitude scores remained relatively moderate across both conditions. Therefore, the absence of significant differences is unlikely to reflect a traditional floor or ceiling effect. Rather, the findings may suggest that sponsor logo density alone was insufficient to generate meaningful variation in overall product evaluations within the present experimental context.

From this perspective, the absence of attitudinal differences should not be interpreted as evidence that visually dense sponsor configurations lack perceptual influence within immersive commerce environments. Rather, the findings suggest that sponsor logo density may exert stronger effects on experiential and cognitive processing during immersive interaction than on broader evaluative judgments following a single AR-based inspection episode. This distinction highlights the importance of considering interaction duration, evaluative task structure, and decision context when examining whether process-level perceptual responses within immersive environments ultimately translate into stable product evaluations (Dhar and Wertenbroch, 2000; Petty and Cacioppo, 1986).

### 4.2 Theoretical and practical implications

The present study contributes to understanding how consumers process visual information density within AR-based retail environments. Specifically, the findings demonstrate that variations in sponsor logo density shape perceptual and cognitive responses during AR-based merchandize evaluation. The findings further indicate that visually information-dense jersey designs systematically influence attentional and arousal responses during immersive product inspection. These findings extend prior sponsorship and consumer research, which has predominantly emphasized sponsor recall or brand attitude formation under passive exposure conditions, by illustrating how visual sponsorship complexity influences earlier stages of perceptual and cognitive processing when products are experienced through immersive AR interaction (Cornwell et al., 2006; Meenaghan, 2001).

The findings additionally contribute to theoretical discussions concerning the relationship between perceptual engagement and broader evaluative judgment within immersive retail environments. Although variations in sponsor logo density produced meaningful differences in attention and arousal, these perceptual and cognitive responses did not translate into significant differences in overall product attitudes following a single AR-based inspection episode. This pattern suggests that visually dense product configurations may exert stronger influence on experiential and cognitive engagement than on broader evaluative orientation during short-term immersive interaction. In this sense, the present findings help clarify how visual complexity may operate differently across stages of consumer response within immersive commerce environments, rather than assuming uniform effects across perceptual, cognitive, and evaluative dimensions (Lavidge and Steiner, 1961).

From a practical perspective, the findings suggest that sponsor logo density should be considered an important visual design characteristic capable of shaping the quality and intensity of immersive product experiences. More specifically, the findings highlight how visual information density may function as a user-centered interface design consideration within immersive commerce systems by influencing perceptual engagement and cognitive activation during interactive product inspection. Within the present study, visually dense jersey configurations increased perceptual activation and altered attentional processing during immersive inspection, indicating that visual information density influences how consumers cognitively engage with products during AR interaction. For sport organizations, sponsors, and digital retailers, these findings suggest that sponsor-rich product presentations may be strategically useful in immersive retail environments where the objective is to stimulate exploration, encourage interaction, or increase experiential engagement during product inspection. Under such conditions, sponsor logos contribute not only to sponsorship visibility but also to the overall perceptual intensity of the immersive retail experience.

At the same time, the absence of attitudinal differences highlights the importance of context-sensitive visual design strategies within immersive commerce systems. When the primary objective involves influencing broader product evaluation or purchase-oriented decision-making, exposure to a single immersive AR interaction may not be sufficient to produce meaningful attitudinal change. Practitioners may therefore benefit from combining immersive product visualization with additional decision-support mechanisms such as repeated exposure opportunities, comparative presentation formats, or purchase-oriented interface features. From this perspective, visually dense sponsor configurations may be more effective during stages of immersive exploration and engagement, whereas visually simplified product presentations may be more appropriate in later stages of the consumer journey that require evaluative clarity and decision-oriented processing. Such an approach may help organizations balance sponsorship visibility, immersive engagement, and user experience optimization across different phases of AR-enabled retail interaction.

### 4.3 Limitations and future research

Several limitations of the present study should be acknowledged, each of which provides meaningful directions for future research within immersive commerce and AR-based product evaluation contexts. First, the participant sample was drawn from a single geographic region, which may limit the broader generalizability of the findings. Perceptions of sponsor density, visual complexity, and tolerance toward commercially saturated apparel designs may differ across cultural environments and sport markets. In particular, familiarity with sponsor-rich uniforms and expectations regarding visual sponsorship integration may vary substantially across regional sport consumption contexts. Future studies could therefore replicate the present design across different national or cultural settings to examine whether perceptual and evaluative responses to visually dense sponsor configurations differ according to regional sponsorship norms or consumer expectations (Hofstede, 2001).

Second, the present study relied primarily on self-reported measures to assess attention, arousal, and evaluative responses during immersive AR interaction. Although these measures are widely used and appropriate for capturing subjective perceptual experiences, they do not directly capture moment-to-moment visual attention or attentional processing during product inspection (Uhm et al., 2020). In addition, participants were intentionally restricted to individuals without prior AR e-commerce experience to ensure a more standardized AR interaction. While this approach minimized familiarity effects, it may also have introduced a novelty effect that elevated baseline arousal levels and influenced participants’ responses to the immersive environment. Furthermore, although participants were allowed to freely rotate and inspect the jerseys, behavioral interaction metrics such as inspection duration were not recorded. Future research could incorporate process-oriented methodologies such as eye-tracking, pupillometry, behavioral interaction measures, or biometric indicators of arousal to more directly examine how visually dense sponsor configurations influence attentional allocation and perceptual activation during immersive AR-based product evaluation. Such approaches may provide a more fine-grained understanding of how visual information density is processed dynamically throughout immersive interaction experiences (Wedel and Pieters, 2008). Future research may also employ longer and more decision-oriented shopping tasks to better capture consumer decision-making processes within AR retail environments.

Third, the experimental design relied on a single between-subject manipulation of sponsor logo density. Although this approach allowed for a controlled examination of the focal variable, it may limit the extent to which broader conclusions regarding consumer processing within AR retail environments can be drawn. Future research could employ more complex experimental designs incorporating additional contextual, technological, or consumer-related factors to provide a more comprehensive understanding of immersive retail experiences. Such approaches may help clarify the boundary conditions under which visual information density influences consumer responses in immersive retail environments. In addition, because the study did not include a non-AR comparison condition, it remains unclear whether the observed effects are specific to AR-based product evaluation or would similarly emerge in other digital retail environments. Future research could directly compare AR and non-AR presentation formats to further clarify the contextual role of immersive technologies in shaping consumer responses.

Fourth, the experimental stimuli used in the present study were limited to professional baseball jerseys, which may constrain the applicability of the findings to other sports or apparel categories. Although baseball represents a relevant context in which sponsor integration is increasing, visual norms surrounding sponsor placement and uniform design differ substantially across sports. For example, visually dense multi-logo uniforms are more established in sports such as soccer and motorsports, potentially leading consumers to process sponsor-rich apparel differently across sport contexts. Future research could extend the present framework to alternative sports, apparel formats, or non-sport retail products to examine whether the observed perceptual and evaluative response patterns generalize across broader immersive commerce environments involving visually information-dense product designs. Lastly, although perceived visual clutter was conceptualized as a subjective perception of visual excessiveness and disruption, some items may also reflect evaluative reactions toward sponsor-rich designs. Future research may further distinguish perceived clutter from evaluative responses using alternative measurement approaches.

## 5 Conclusion

The present study contributes to understanding how visual information density influences perceptual and cognitive responses within AR-based product evaluation contexts. Using professional sport jerseys as a context for immersive interaction, the findings indicate that visually dense sponsor configurations shape attention, arousal, and perceptions of visual clutter during interactive inspection, while broader evaluative attitudes remain comparatively stable. The findings suggest that consumer responses within AR-based retail environments may be shaped not only by the immersive presentation context but also by the structural complexity of the visual information embedded within products.

## Data Availability

The raw data supporting the conclusions of this article will be made available by the authors, without undue reservation.

## Appendix

**Table:**

Perceived clutter	While inspecting the jersey in the AR environment… (1 = *Strongly disagree*, 7 = *Strongly agree*) These sponsor logos made up a lot of the visual clutter. There were too many sponsor logos. These sponsor logos took up too much space on the jersey. These sponsor logos were distracting. These sponsor logos were irrelevant to the jersey design. These sponsor logos were annoying.
Arousal	While viewing the jersey in the AR environment, I felt… (*Responses range from 1 to 7, with 1 indicating the left word and 7 indicating the right word*.) Sleepy – Aroused Calm – Excited Relaxed – Stimulated Dull – Jittery Sluggish – Frenzied
Attention	While viewing the jersey in the AR environment, how closely did you attend to it? (*Select one number from 1 to 7 that best represents your experience.*) I did not pay attention at all – I paid very close attention I attended to the jersey very vaguely – I attended to the jersey very intensely I was hardly attentive while viewing the jersey – I was very attentive while viewing the jersey
Attitude	After viewing the jersey in the AR environment, my overall attitude toward the jersey was… (*Responses range from 1 to 7, with 1 indicating the left word and 7 indicating the right word*.) Negative – Positive Bad – Good Unfavorable – Favorable
